# Deep learning based two-way feature depiction model for brain tumor detection

**DOI:** 10.1371/journal.pone.0344291

**Published:** 2026-07-02

**Authors:** Shabana Urooj, Kiran Napte, Najah Alsubaie

**Affiliations:** 1 Department of Electrical Engineering, College of Engineering, Princess Nourah bint Abdulrahman University, Riyadh, Saudi Arabia; 2 Department of Electronics and Telecommunication, PCET’s Pimpri Chinchwad College of Engineering and Research, Ravet, Pune, India; 3 Department of Computer Sciences, College of Computer and Information Sciences, Princess Nourah bint Abdulrahman University, Riyadh, Saudi Arabia; Dayananda Sagar University, INDIA

## Abstract

Brain tumors are one of the most fatal disorders that cause one of the highest mortalities in the world. Gliomas are the most common primary brain tumors originating from glial cells in the central nervous system. Traditionally, a tissue sample is extracted and examined for its genetic and characteristic properties. This method is invasive, painful, and takes a longer period to produce results. Various automatic Deep learning (DL) based schemes have been presented for the brain glioma detection, but they lack due to poor explainability, lower generalization, poor feature depiction, class imbalance problem and lower detection rate. This paper presents a deep learning based brain tumor detection using two way feature depiction model (TWFDM) that combines the 2D-Deep Convolution Neural Network (DCNN) and 1D-DCNN. The 2D-DCNN accepts the raw MRI images and the 1D-DCNN accepts the handcrafted local binary pattern (LBP), gray level cooccurrence matrix (GLCM), and Histogram of Oriented Gradient (HOG) features. Furthermore, improved particle swarm optimization (IPSO) is used for feature selection to minimize the computational complexity of the TWFDM system. The proposed TWFDM achieves an overall accuracy of 96.25%, a recall of 96.34%, a precision of 96.31%, and an F1-score of 96.32% on the Brain MRI dataset for four-class classification, representing an important improvement over traditional techniques.

## 1 Introduction

Brain tumors represent one of the most life-threatening neurological disorders caused by the uncontrolled proliferation of abnormal cells within the brain or skull. They are broadly categorized into primary and secondary types. Primary brain tumors, which constitute nearly 70% of all cases, originate within the brain and remain localized. In contrast, secondary brain tumors develop in other organs—such as the lungs, breasts, or kidneys—before metastasizing to the brain [1]. The three most prevalent types of brain tumors include gliomas, meningiomas, and pituitary tumors. Among them, gliomas—originating from the uncontrolled growth of glial cells, which make up nearly 80% of brain tissue—are considered the most aggressive and fatal. Meningiomas, in contrast, form in the meninges, the protective membranes of the brain and spinal cord, while pituitary tumors arise in the pituitary gland, which regulates essential hormones [2]. Although pituitary tumors are generally benign, they can cause severe hormonal imbalances and even permanent vision loss. Consequently, early and accurate diagnosis of brain tumors is vital to prevent irreversible complications and improve patient outcomes [3].

To identify and characterize brain tumors, medical professionals rely on several imaging modalities, including Computed Tomography (CT), Magnetic Resonance Imaging (MRI), and ultrasound [4]. Among these, MRI is the most preferred due to its non-invasive nature and the absence of harmful ionizing radiation. MRI scans can generate detailed views of soft tissues using various contrast settings, such as T1, T2, and FLAIR sequences, enabling clearer visualization of abnormalities [5]. However, accurate tumor identification remains difficult because tumor shape, size, severity, and location can vary greatly. Traditionally, radiologists manually delineate tumor boundaries after visually analyzing MRI images. Yet, tumor edges often blend with surrounding healthy tissues, making manual inspection labor-intensive and prone to human error. The accuracy of this process heavily depends on the radiologist’s experience and expertise [6]. Moreover, the subtle grayscale variations in MRI images are not easily perceptible to the naked eye, complicating the differentiation between malignant and benign lesions.

To overcome these challenges, researchers have proposed systematic approaches using MRI-based deep learning frameworks that analyze tumor depth, size, and heterogeneity, particularly on T2-weighted images (T2WI), to improve diagnostic accuracy. In studies such as [7,8], various DCNN and machine learning (ML) classifiers were tested on three benchmark datasets—BT-small-2c, BT-large-2c, and BT-large-4c. Experimental findings revealed that DenseNet-169 performed effectively on small, binary datasets, while an ensemble of DenseNet-169, InceptionV3, and ResNeXt-50 was more suitable for large, binary-class datasets. For multi-class classification (normal, glioma, meningioma, pituitary), combining DenseNet-169, ShuffleNetV2, and MnasNet yielded the best outcomes. However, traditional ML-based models have a major drawback: manual feature extraction, where relevant features must first be defined and extracted from the training data [9].

Brain tumor classification approaches can generally be divided into two main paradigms: ML and DL. ML-based systems depend heavily on handcrafted feature extraction and manual segmentation, both of which are time-consuming and error-prone processes. These systems often require domain experts to identify the most effective combination of segmentation and feature extraction techniques, leading to inconsistent performance across different datasets [10]. In contrast, deep learning models, particularly Convolutional Neural Networks (CNNs), have demonstrated superior capability in automating these tasks. CNNs can automatically learn both low-level and high-level representations from data, eliminating the need for manual intervention. This automation, combined with their weight-sharing structure and robust generalization ability, has made CNN-based models the leading choice for medical image analysis. As a result, deep learning has garnered significant attention from researchers and clinicians alike, revolutionizing the field of computer-aided brain tumor diagnosis [11,12].

This paper presents brain tumor detection using TWFDM for multi-class brain tumor detection. The major contributions of the article are summarized as follows:

- Multi-class brain tumor detection using TWFD that combines 2D-DCNN and 1D-DCNN. The 2D-DCNN accepts the brain MRI images to describe the spatial correlation in local region of the images. The 1D-DCNN accepts the handcrafted features using local binary pattern (LBP) to provide local texture variations, grey level co-occurrence matrix (GLCM) to offer global texture variation and Histogram of Oriented Gradients (HOG) to describe the shape attributes of the brain in MRI.
- Further, improved PSO with position encouraging scheme is utilized for the feature selection to lessen the computational complexity and lowering the trainable parameters of 1D-DCNN.
- The effectiveness of the system is assessed on the public brain tumor MRI dataset (Kaggle) based on various performance metrics.

The remaining article is arranged as follows: Sect 2 provides the related work of the brain tumor detection systems. Sect 3 offers the TWFDM details. Sect 4 delivers the discussions on the simulation results. Lastly, Sect 5 offers the conclusions and future scopes of the work.

## 2 Related work

Recent advancements in brain cancer detection using deep learning have demonstrated remarkable improvements in accuracy, robustness, and efficiency. Sarala et al. [8] proposed an edge-preserving image fusion approach integrated with a dual CNN and Histogram-Density Segmentation Algorithm (HDSA). Their model enhanced tumor localization and classification accuracy for glioma detection on the BraTS-IXI dataset, although it required high computational power and lacked specific performance metrics. Similarly, Saravanan et al. [9] developed a Convolutional Deep Learning model with Neighboring Network Limitation (CDBLNL), which utilized metadata-based vector encoding to achieve superior results on BRATS and REMBRANDT datasets. Despite its strong classification ability, the model’s complex structure led to high dimensionality issues.

Ullah et al. [10] introduced TumorDetNet, a 48-layer CNN that employs LReLU/ReLU activations and dropout regularization, achieving up to 99.83% accuracy for binary classification and 99.27% for multi-class classification using Kaggle MRI datasets. Although highly robust, it was computationally expensive. Abdusalomov et al. [11] applied YOLOv7 with transfer learning, achieving 99.5% accuracy while providing strong tumor localization; however, small tumor detection remained a challenge. Mahmud et al. [12] used an ensemble CNN with VGG16, achieving around 98% accuracy on 7,023 MRI scans; however, the ensemble structure increased model complexity and risked overfitting.

Mathivanan et al. [13] applied transfer learning using architectures such as ResNet-152, VGG-19, DenseNet-169, and MobileNetv3, achieving a maximum accuracy of 99.75% with MobileNetv3 on the Kaggle dataset. Although this approach offered high generalization, it demanded significant GPU resources. Rizwan et al. [14] proposed a Gaussian CNN (GCNN) capable of multi-class and grade-level glioma detection, yielding an accuracy of up to 99.8%. However, the limited dataset size affected performance for higher-grade gliomas. Bhimavarapu et al. [15] combined Improved Fuzzy C-Means clustering with Extreme Learning Machine (ELM) for glioma detection, achieving 99.75% accuracy, yet its sensitivity to noise impacted reliability.

The Deep Learning-based Brain Tumor Detection and Classification using MRI (DLBTDC-MRI) system, developed by Mohan et al. [16] utilizes deep learning features and manually crafted image descriptors to enhance diagnosis accuracy. The model utilized Cuckoo Search Optimization (CSO) to determine suitable tumor area thresholds for segmentation. MRI noise was removed, and picture clarity was improved by adaptive fuzzy filtering (AFF). To enhance tumor characterisation, CNN backbone deep features were combined with handmade texture and shape descriptors. Hybrid CNNs exhibit better feature discrimination and classification accuracy compared to standard CNNs. Its capacity to capture both low-level structural features and high-level contextual information makes it a valuable tool for tumor depiction. After integrating various feature extraction and optimization modules, the framework became computationally demanding, requiring significant processing time and memory, which limited its scalability for large datasets or real-time diagnosis. Khan et al. [17] suggested a 23-layer CNN with VGG16-based transfer learning to identify and classify brain cancers. Using pre-trained weights from VGG16, transfer learning improved convergence and performance on MRI images of glioma, meningioma, and pituitary tumors, particularly when limited data was available. Great classification precision was achieved with 100% accuracy on small datasets and 97.8% on larger ones. Strong feature extraction and resilience across binary and multiclass settings were the main advantages of their model. Due to the small sample size and deep network design, the system overfitted and could not generalize on unseen data.

Hybrid Transfer Learning (HTL) was used by Golkarieh et al. [18] to improve tumor classification using VGG19, ResNet50, InceptionV3, and EfficientNetV2. The ensemble was trained using MRI data from patients with glioma, meningioma, pituitary tumors, neurocytoma, and abscesses. Due to its compound scaling mechanism, which balances depth, breadth, and resolution to enhance performance and efficiency, EfficientNetV2 proved to be the best performer after a rigorous comparison. Across tumor classifications, the hybrid model performed better feature extraction and classification. It demonstrates how multi-model fusion generalizes effectively across diverse datasets. The primary drawbacks were extensive training time and high computing cost, which might restrict its use in clinical situations where speedy decision-making is critical. Saeedi et al. [19] proposed a 2D CNN/CAE brain tumor detection and classification system. CAE performed unsupervised feature learning, while CNN classified. Automatic picture feature extraction was possible without human preprocessing using this two-step procedure. The CNN and CAE models performed well for simpler and well-structured tumors, achieving 96.47% and 95.63% accuracy, respectively, on glioma, meningioma, pituitary, and normal MRI images. This work is essential because of its computational simplicity and efficient training process, making it practical. The performance worsened for complex and diverse tumors, demonstrating inadequate robustness.

Rahman and Islam [20] developed a Parallel Deep Convolutional Neural Network (PDCNN) architecture to enhance tumor classification by extracting local and global information. The parallel CNN branches gathered fine-grained texture patterns and global contextual data from MRI images. Resizing, grayscale conversion, and data supplementation increased model generalization. The PDCNN outperformed multiple CNNs with 98.12% accuracy on benchmark datasets. Parallelization increased feature variety and decreased duplication, making this technique strong. Real-time medical applications were hindered by the model’s computational complexity and resource needs. Qureshi et al. [21] proposed an Ultra-Light Deep Learning Architecture (UL-DLA) that utilizes GLCM texture characteristics and SVM classification. This hybrid system sought diagnostic precision and real-time performance. The lightweight CNN retrieved shallow features, whereas GLCM caught local intensity fluctuations using statistical texture descriptors. These characteristics were categorized using an optimal SVM. The UL-DLA model demonstrated high accuracy, achieving 99.23% for portable diagnostic devices. The lightweight design makes it ideal for edge devices and low-power electronics. When applied to new datasets, the model required fine-tuning, which limited its versatility. Asiri et al. [22] created a ResNet50–U-Net hybrid architecture that incorporated segmentation and classification. U-Net segmented tumor pixels, whereas ResNet50 classified them. The model accurately delineated glioma areas in MRI images with an Intersection over Union (IoU) of 0.91 and a Dice Similarity Coefficient (DSC) of 0.95. Its end-to-end capacity to accurately detect and categorize cancers makes this technique important. It only detected gliomas, limiting its application to other tumors.

Ullah et al. [23] employed transfer learning to enhance tumor classification accuracy using various pre-trained models, including InceptionResNetV2, ResNet, DenseNet, and MobileNetV2. The model outperformed various hybrid models with 98.91% accuracy using pre-trained feature representations. Due to deep hierarchical features and transfer learning flexibility, it trained models faster than from scratch. However, the framework was hard to grasp, making it hard to determine which factors influenced categorization. Priya and Vasudevan [24] created a Hybrid AlexNet–GRU model that combines convolutional spatial learning with Gated Recurrent Units for temporal sequence modeling. To maintain texture and structure, MRI images were denoised using a non-local means filter. AlexNet gathered spatial information from photos and sent them to GRU layers to capture slice dependencies. The system modeled spatial-temporal connections with 97% accuracy. Gradient vanishing and overfitting plagued the model, especially on small datasets. Finally, Yildirim et al. [25] proposed a hybrid EfficientNetB0–ShuffleNet–SVM model for brain tumor classification. CNN backbones EfficientNetB0 and ShuffleNet extracted features, whereas SVM classified. The technique achieved 95.4% accuracy on bespoke MRI datasets using lightweight networks and SVMs. The model’s balanced computational efficiency and performance make it appropriate for clinical use under hardware constraints. However, its accuracy was somewhat lower than that of pure deep learning models, suggesting the need for improvements in feature fusion and optimization.

Thus, these studies highlight a clear trend toward hybrid deep learning and transfer learning architectures that combine CNNs with optimization or auxiliary models to enhance accuracy and generalization for brain cancer detection [26,27]. However, challenges such as computational complexity, interpretability, and dataset diversity remain key areas for future research [28–30]. The significant gaps identified are summarized as follows:

- Many deep learning models require high computational resources, limiting real-time clinical use.
- Overfitting on small datasets reduces generalization to unseen MRI scans.
- Accurate detection of small or subtle tumors remains challenging.
- Hybrid and ensemble models increase complexity, training time, and memory usage.
- MRI noise and artifacts negatively affect model robustness and accuracy.
- Most studies focus on a single tumor type, limiting broader applicability.
- Lack of interpretability hinders clinical trust in deep learning models.
- Lightweight models for edge devices often compromise detection accuracy.
- Diverse datasets and evaluation metrics make benchmarking difficult.
- Existing models face scalability issues for large or multi-center datasets.

## 3 Methodology

**Fig 1:**
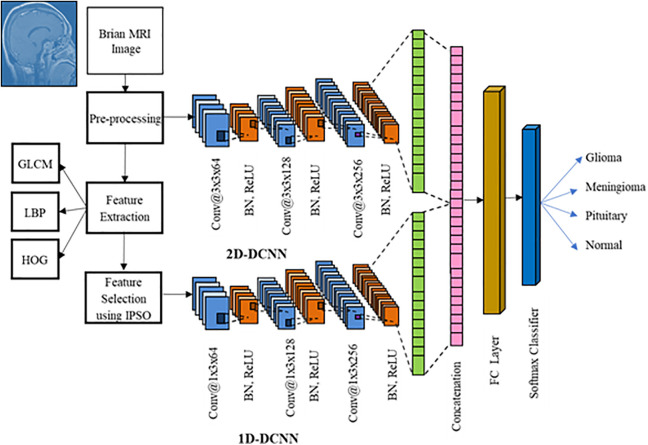
Flow diagram of the proposed TWFDM for brain tumor detection system.

Fig 1 illustrates a hybrid deep-learning-based brain tumor classification framework that integrates handcrafted and deep features to improve diagnostic accuracy. The process begins with pre-processing, where MRI images are enhanced and normalized to remove noise and improve clarity. Furthermore, feature extraction is performed through two parallel paths: 2D-DCNN and 1D-DCNN. In the first path, MRI images are processed using a 2D-DCNN with three convolution layers (64, 128, 256 filters, 3 × 3 kernels, stride 1, zero padding), followed by ReLU and batch normalization to capture spatial structure and morphology. In the second path, handcrafted features such as LBP, GLCM, and HOG are extracted to capture texture and shape information and then passed through a 1D-DCNN with three convolution layers (64, 128, 256 filters, 1 × 3 kernels, stride 1), also followed by BN and ReLU. The outputs from both networks are flattened, concatenated, and fed into a fully connected layer, followed by a final softmax classifier to detect the brain tumor.

Next, feature extraction is performed through two parallel pathways. The first parallel path uses MRI images and a 2D-DCNN to depict spatial correlations in brain structure and morphology. The 2D DCNN consists of three convolutional layers with 64, 128, and 256 filters, each using a 3x3 kernel, a stride of 1 pixel, and zero padding at the borders. The convolutional layer is followed by a ReLU layer to enhance nonlinearity and by batch normalization (BN) to accelerate training. Furthermore, LBP, GLCM, and HOG-based handcrafted features are extracted to capture texture and shape attributes of the MRI images and are fed into a 1D-DCNN to improve the hierarchical depiction of the features. The 1D-DCNN consists of three layers: 1D convolutional layers with 64, 128, and 256 filters, each using a 1x3 kernel and a stride of 1 pixel. A BN and a ReLU layer follow the convolution layers. The outputs of the last layers of the 2D-DCNN and 1D-DCNN are flattened and concatenated. Further, a fully connected layer is employed for improving the connectivity in the features and a softmax classifier for detecting the brain tumor.

### 3.1 LBP, GLCM and HOG Feature Extraction

#### A. LBP.

The LBP provides the texture description over the local 3 × 3 region of the images. The neighboring pixels of the local window are compared with the centered pixel; if the value of the pixel is greater than the centering pixel, then it is considered as binary ‘1’, otherwise ‘0’. The local window provides a binary pattern of eight bits, which is converted into a decimal equivalent and replaced at the centered pixel position. The LBP descriptor captures local variations in MRI images resulting from changes in the brain’s structure and morphological properties. The histogram of LBP provides the 256 features, which are scale-invariant and rotation-invariant. The LBP process equation is given in Eq 1, where x and y depict row and column position, c indicates the center position in the window, and LBP denotes the LBP pattern.


$$\begin{array}{c} LBP(x,y)=\left\{ \begin{array}{cc} \text{1},\; & im(x,y)>im(c,c)\\ \text{0},\; & \;otherwise \end{array}\right.\; \end{array}$$ (1)

#### B. GLCM.

The GLCM provides the global texture depiction of the brain MRI images using 12 features such as Contrast, Correlation, Energy (ASM), Homogeneity (Inverse Difference Moment), Entropy, Variance, Dissimilarity, Cluster Shade, Cluster Prominence, Maximum Probability, and Sum Entropy. These features quantify relationships between pixel intensities, capturing variations, patterns, and irregularities in tissue structure. For example, contrast and dissimilarity highlight intensity variations and tumor boundaries, while correlation measures the linear dependency of gray levels, helping differentiate homogeneous healthy tissue from heterogeneous tumor regions. Energy and homogeneity assess texture uniformity and smoothness, indicating abnormal regions, whereas entropy and sum entropy quantify randomness, which is often increased in tumor areas due to irregular growth. Higher-order features like cluster shade and cluster prominence capture asymmetry and peakedness in intensity distributions, reflecting irregular tumor shapes. Features such as maximum probability and variance further characterize dominant textures and intensity dispersion. Overall, these 12 GLCM features provide a comprehensive representation of texture, enabling effective differentiation of tumor tissue from normal brain tissue in MRI scans.

#### C. HOG.

The HOG provides a shape description of the MRI images to depict changes in brain morphology and structure due to the tumor. The HOG features are computed over a cell size of 8 × 8 pixels, a block size of 2 × 2 cells with 50% overlapping, 9 bin orientation, and an image size of 256 × 256 pixels. The HOG provides the 34596 features. The final feature vector consists of 34864 features encompassing 12 GLCM features, 256 LBP features, and 34596 HOG features.

### 3.2 IPSO for feature selection

The initial population of the PSO depicts the possible combinations of the features to be chosen from the total features ($N_{feat})$. Each row depicts the indices of the features representing the LBP, GLCM, and HOG. The PSO population is provided in Eq 2 and created using Eq 3, where P denotes the PSO population, p indicates the feature index, n denotes the total features to be chosen, N indicates the number of particles, LB and UB are the lower bound (1) and upper bound ($N_{feat}$).


$$\begin{array}{c} P=[\begin{array}{cccc} p_{\text{1}\text{1}} & p_{\text{1}\text{2}} & \cdots & p_{\text{1}n}\\ p_{\text{2}\text{1}} & p_{\text{2}\text{2}} & \cdots & p_{\text{2}n}\\ \vdots & \vdots & \ddots & \vdots \\ p_{N\text{1}} & p_{N\text{2}} & \cdots & p_{N\text{2}} \end{array}] \end{array}$$ (2)

$$\begin{array}{c} p_{i}=LB+rand(\text{1})*(UB-LB) \end{array}$$ (3)

Further, the fitness of each particle is computed using Eq 4, which is based on the ratio of intra-class to inter-class variance of the features.


$$\begin{array}{c} Fitness=\frac{\delta_{intraclass}}{\delta_{interclass}} \end{array}$$ (4)

The PSO updates the particle’s position by adjusting its velocity if the fitness is not optimal. The IPSO considers the position encouraging exploration that helps improve the population’s search space. The novel velocity updation is provided in Eq 5.


$$\begin{array}{c} v_{i}(t+\text{1})=w\cdot v_{i}(t)+c_{\text{1}}\cdot r_{\text{1}}\cdot (\overset{best}{\underset{i}{p}}-x_{i}(t))+c_{\text{2}}\cdot r_{\text{2}}\cdot (g^{best}-P_{i}(t))+c_{\text{3}}\cdot r_{\text{3}}\cdot (n_{i}(t)-P_{i}(t)) \end{array}$$ (5)

Where, $v_{i}(t)$ denotes particle velocity of particle i at iteration t, w stands for inertia weight, $c_{\text{1}},c_{\text{2}},and\;c_{\text{3}}$ symbolizes acceleration coefficients for personal, global, and novelty influences, $r_{\text{1}},r_{\text{2}},\;and\;r_{\text{3}}$ signifies random numbers uniformly distributed in [0,1], $\overset{best}{\underset{i}{p}}$ denotes the personal best position of the particle i, $g^{best}$ is the global best position among all particles, $n_{i}(t)$ symbolizes a position encouraging the exploration coefficient and $P_{i}(t)$ signifies the current position of the particle i.

The position encouraging coefficient is computed using Eq 6, which considers k neighboring particles of the particle to generate a diverse population by repelling swarms from crowded regions.


$$\begin{array}{c} n_{i}(t)=\frac{\text{1}}{k}\sum_{j=\text{1}}^{k}\left(P_{i}(t)-P_{j}(t)\right) \end{array}$$ (6)

Here, k denotes number of neighboring particles and $P_{j}(t)$: positions of neighboring particles. The final particle position is updated using Eq 7.


$$\begin{array}{c} P_{i}(t+\text{1})=P_{i}(t)+v_{i}(t+\text{1}) \end{array}$$ (7)

The algorithm for the IPSO is given as follows:

**Algorithm 1.** Improved Particle Swarm Optimization (PSO)

Input: Population size N, maximum iterations T, inertia weight w, acceleration coefficients $c_{\text{1}},c_{\text{2}}$

Output: Global best solution <<Eqn28»

1. Initialize positions $P_{i}$and velocities $v_{i}$for all particles <<Eqn31»

2. Evaluate the fitness of each particle using Eq 4.

3. Set personal best $p_{best,i}=x_{i}$for each particle.

4. Determine global best $g_{best}$among all $p_{best,i}$.

5. For iteration t=1to T, do

  5.1 For each particle i, do

    a) Update velocity using Eq 5 using the position encouraging scheme.

    b) Update position using Eq 7.

    c) Evaluate new fitness of particle i using equation 4.

    d) Update $p_{best,i}$if new position is better.

  5.2 Update $g_{best}$from all $p_{best,i}$.

6. End For

7. Return $g_{best}$.

## 4 Experimental results and discussions

### 4.1 Dataset

**Fig 2:**
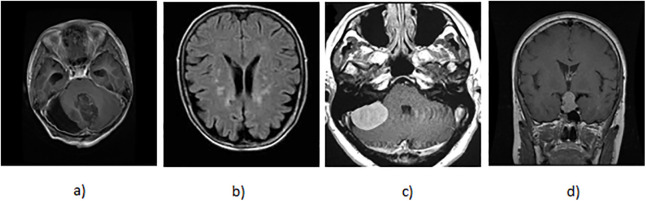
Samples of dataset a) Glioma b) Normal c) Meningioma d) Pituitary.

The effectiveness of the tumor detection is evaluated on the public brain tumor MRI dataset available on Kaggle [31]. The brain tumor MRI dataset comprises brain MRI images in four classes: glioma, meningioma, pituitary, and normal. The dataset details are summarized in Table 1. All images are resized to a 256 × 256 pixel resolution. The samples of the dataset are provided in Fig 2.

**Table 1:** Brain tumor MRI Dataset details.

Class	Training Images (70%)	Testing Images (30%)
Glioma	1321	300
Meningioma	1339	306
Pituitary	1457	300
Normal	1595	405

### 4.2 Discussions on results

**Fig 3:**
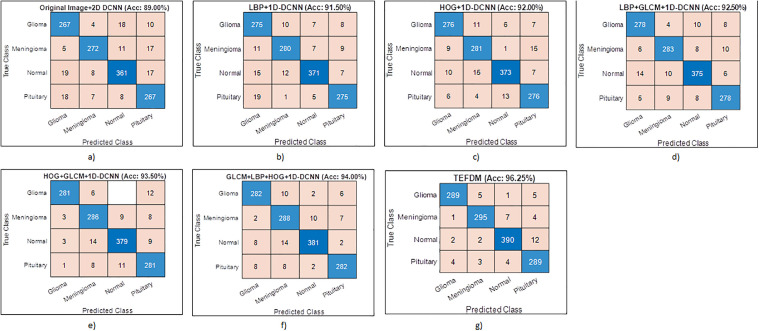
Confusion matrices for various feature depiction and models.

The confusion matrices for the various feature depiction schemes and DL models is shown in Fig 3. Table 2 provides the comparative results for the various models and feature depiction schemes. The Original Image + 2D DCNN served as the baseline model, achieving an average accuracy of 89.00%, recall of 89.08%, precision of 89.16%, and F1-score of 89.12%. These nearly balanced metrics indicate consistent but moderate learning capability using only raw MRI images. When texture information from Local Binary Patterns was added in the LBP + 1D-DCNN, performance improved to an average accuracy of 91.54%, recall of 91.69%, precision of 91.58%, and F1-score of 91.63%. This represents a 2.54% improvement in accuracy and about 2.5% increase in recall and F1-score, mainly due to better texture encoding that enhanced the distinction of Normal and Pituitary tumor types. Similarly, the HOG + 1D-DCNN model achieved an average accuracy of 91.99%, recall of 91.93%, precision of 91.80%, and F1-score of 91.84%, confirming that gradient-based features effectively captured edge and shape patterns of tumors, albeit with only a 0.45% increase in accuracy over the LBP-based model. Further integrating Gray-Level Co-occurrence information into LBP + GLCM + 1D-DCNN resulted in an accuracy of 92.60%, recall of 92.60%, precision of 92.47%, and F1-score of 92.53%. This marks an overall 3.6% improvement from the baseline and demonstrates the added value of spatial co-occurrence statistics.

**Table 2:** Comparative results for various feature depiction schemes.

Model	Class	Accuracy	Recall	Precision	F1-score
Original Image + 2D DCNN	Glioma	89.00	89.00	88.70	88.85
	Meningioma	89.00	89.18	89.18	89.18
	Pituitary	89.00	89.00	90.51	89.75
	Normal	89.01	89.14	88.26	88.70
	Average	89.00	89.08	89.16	89.12
LBP + 1D-DCNN	Glioma	91.37	91.97	90.16	91.06
	Meningioma	91.50	91.50	91.21	91.35
	Pituitary	91.67	91.67	91.97	91.82
	Normal	91.60	91.60	92.98	92.29
	Average	91.54	91.69	91.58	91.63
HOG + 1D-DCNN	Glioma	92.00	92.00	90.20	91.09
	Meningioma	92.00	91.83	94.93	93.36
	Pituitary	92.00	92.00	87.90	89.90
	Normal	91.97	91.87	94.19	93.02
	Average	91.99	91.93	91.80	91.84
LBP+GLCM + 1D-DCNN	Glioma	92.67	92.67	89.68	91.15
	Meningioma	92.48	92.48	93.71	93.09
	Pituitary	92.67	92.67	92.05	92.36
	Normal	92.59	92.59	94.46	93.52
	Average	92.60	92.60	92.47	92.53
HOG+GLCM + 1D-DCNN	Glioma	93.88	93.98	92.74	93.36
	Meningioma	93.46	93.46	93.46	93.46
	Pituitary	93.37	93.67	91.83	92.74
	Normal	93.58	93.58	95.95	94.75
	Average	93.57	93.67	93.50	93.58
GLCM+LBP + HOG + 1D-DCNN (2000 features)	Glioma	94.00	94.00	92.76	93.38
	Meningioma	94.11	94.12	95.05	94.58
	Pituitary	94.00	94.00	92.76	93.38
	Normal	94.00	94.31	95.49	94.89
	Average	94.03	94.11	94.02	94.06
TWFDM	Glioma	96.33	96.33	96.01	96.17
	Meningioma	96.21	96.41	95.78	96.09
	Pituitary	96.00	96.33	96.66	96.49
	Normal	96.46	96.30	96.77	96.53
	Average	96.25	96.34	96.31	96.32

**Fig 4:**
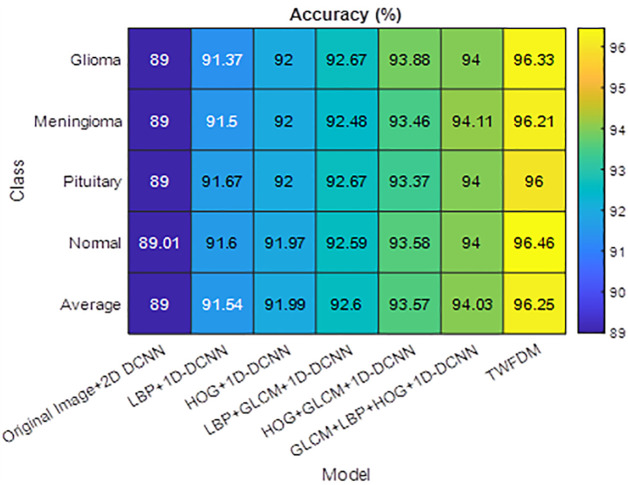
Heatmap for the accuracy of tumor detection.

**Fig 5:**
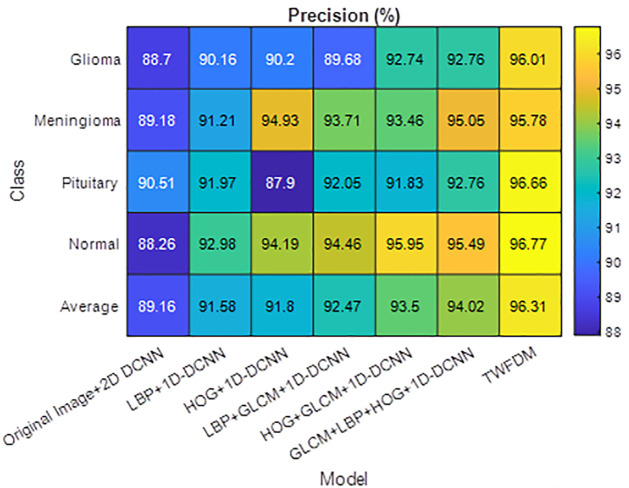
Heatmap for the precision of tumor detection.

**Fig 6:**
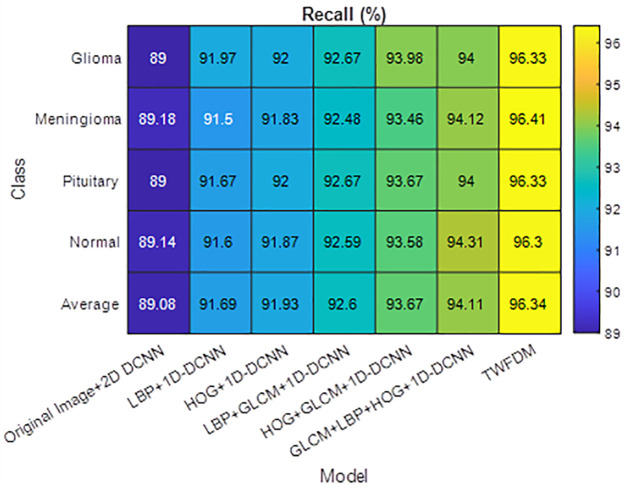
Heatmap for the recall of tumor detection.

The experimental results are obtained for the 2000 features chosen using IPSO. The HOG + GLCM + 1D-DCNN combination reached 93.57% accuracy, 93.67% recall, 93.50% precision, and 93.58% F1-score, yielding an additional 1% gain over the previous hybrid. The improvement demonstrates that merging gradient and spatial texture features enhances the robustness of tumor boundary recognition. When all descriptors were combined in GLCM + LBP + HOG + 1D-DCNN, the system achieved an average accuracy of 94.03%, a recall of 94.11%, a precision of 94.02%, and an F1-score of 94.06%. Compared with the baseline, this reflects a 5.03% rise in accuracy and demonstrates strong feature complementarity across all four tumor classes. Combining both texture and spatial information in LBP + GLCM + 1D-DCNN increased the average accuracy to 92.6%, representing a 4% improvement over the baseline and highlighting the significance of spatial co-occurrence features. The HOG + GLCM + 1D-DCNN model achieved 93.6% accuracy, with precision and recall above 93%, reflecting a 5.6% performance improvement attributed to the synergistic fusion of texture and gradient. Adding multiple descriptors simultaneously in GLCM, LBP, HOG, and 1D-DCNN led to further refinement, achieving 94.1% recall, 94.0% precision, and 94.1% F1-score, indicating consistent classification of all tumor types and robust generalization. The proposed TEFDM model achieved the highest performance, with an accuracy of 96.25%, a recall of 96.34%, a precision of 96.31%, and an F1-score of 96.32%. This represents a 7.25% accuracy improvement and approximately 7.2% enhancement in recall and F1-score compared with the 2D DCNN baseline. For individual tumor categories, glioma achieved a 96.33% F1-score, Meningioma 96.09%, Pituitary 96.49%, and Normal 96.53%, indicating balanced and reliable classification across all types. Visualization of results are shown in Fig 4, Fig 5, and Fig 6.

**Fig 7:**
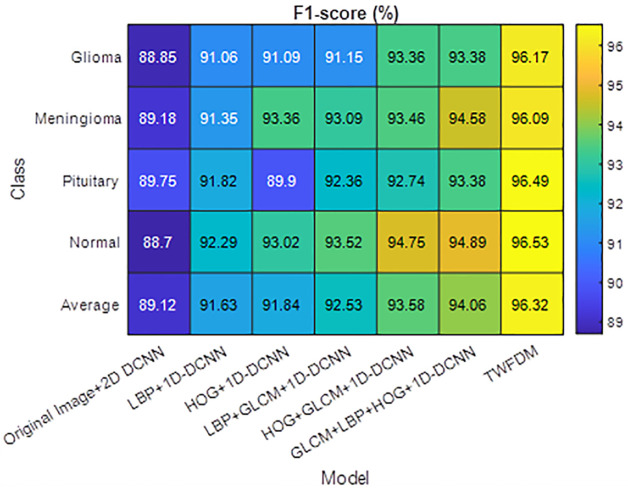
Heatmap for the F1-score of tumor detection.

The receiver operating characteristic (ROC) curve of TWFDM is shown in Fig 7, which illustrates how well the brain tumor detection model distinguishes between tumor and non-tumor cases and the trade-off between sensitivity and specificity.

We have evaluated the effectiveness of the different training and testing sample ratios. It is observed that 70% of the data for training provides better stability in the training model, and 30% of the data provides enough samples for validating the model’s performance. It provides 92.15% accuracy for 40%, 93.75% for 50%, 94.50% for 60%, 96.25% for 70%, 95% for 80%, and 95.45% for 90% training samples as shown in Table 3.

**Table 3:** Performance of TWFDM for different training and testing splits.

Training Samples (%)	Testing Samples (%)	Accuracy (%)
40	60	92.15
50	50	93.75
60	40	94.50
70	30	96.25
80	20	95.00
90	10	95.45

**Fig 8:**
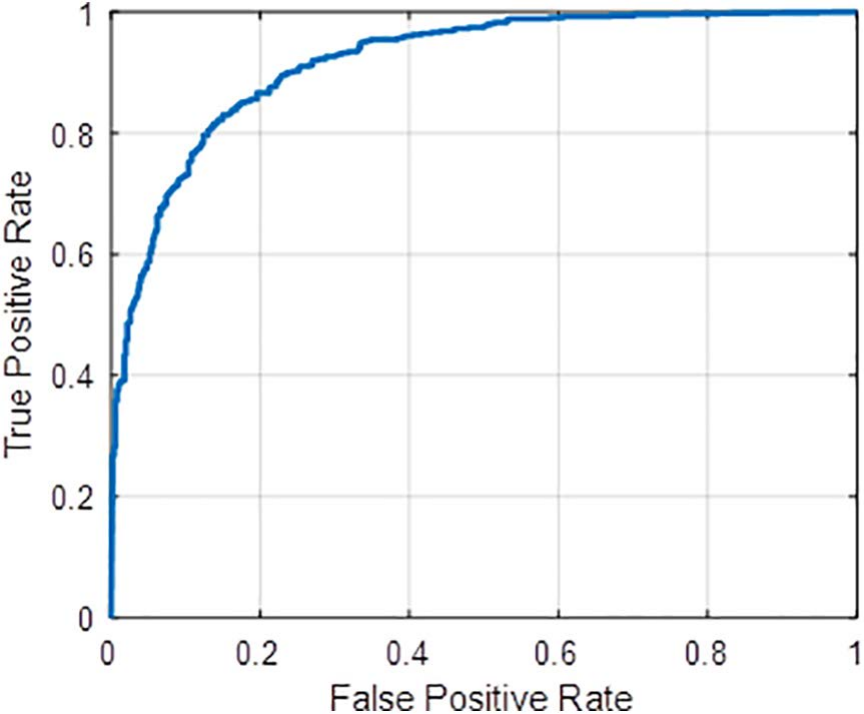
ROC for TWFDM-based brain tumor detection.

**Fig 9:**
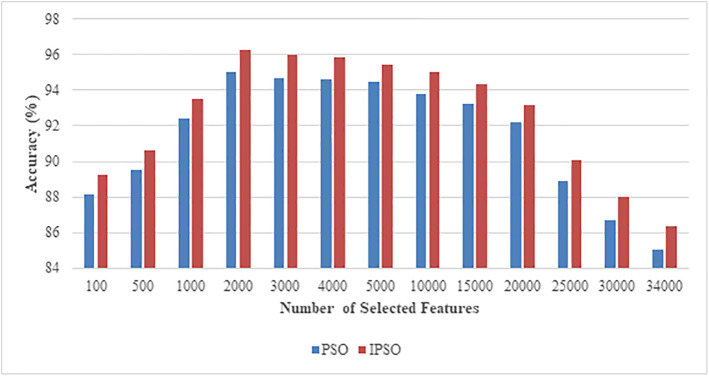
Comparison of PSO and IPSO-based feature selection for brain tumor detection using TWFDM.

The IPSO outcomes for different feature sets, based on overall accuracy, are shown in Fig 8. The IPSO achieves higher accuracy (95.02%) than traditional PSO (95.02%) for 2000 features, as the position-encouraging scheme helps update the population towards an optimal solution in a stepwise manner. Random updates in the PSO lead to a poor search space and lower accuracy in tumor detection. With more features, accuracy decreases due to feature redundancy, which reduces discriminability (Fig 9).

We have analyzed the confidence interval, standard deviation, and average training time of the proposed TWFDM for running the model 10 times to analyze the statistical behavior of the model. The TWFDM-IPSO model shows the tightest and highest confidence interval [0.9507,0.9713], indicating more consistent and superior performance compared to the baseline TWFDM and TWFDM-PSO models. The low standard deviations across models (ranging from 0.0134 to 0.0166) suggest that the performance remains stable across multiple runs, with minimal variation. Additionally, the optimized versions TWFDM-PSO and TWFDM-IPSO achieve faster training times than the original TWFDM method, showing that feature selection not only boosts accuracy but also improves computational efficiency as shown in Table 4.

**Table 4:** Statistical analysis of TWFDM-based brain tumor detection.

Model	Confidence Interval	Standard Deviation	Average Training Time (s)
TWFDM	[0.8934, 0.9100]	0.0134	1835 sec
TWFDM-PSO	[0.9356, 0.9524]	0.0136	1643 sec
TWFDM-IPSO	[0.9507,0.9713]	0.0166	1572 sec

The comparative evaluation of different brain tumor detection methodologies on the same benchmark dataset clearly demonstrates the superiority of the proposed TWFDM model as given in Table 5. Among existing works, Saeedi et al. (2023) achieved an accuracy of 95.65% using a 2D CNN combined with a CAE, demonstrating strong representation learning but moderate generalization on unseen data. Similarly, Rahman and Islam (2023) employed a Parallel Deep CNN (PDCNN) approach, achieving 94.25% accuracy, indicating effective spatial feature extraction but slightly lower robustness than hybrid or transfer learning models. The method presented by Qureshi et al. (2022) integrated the Ultra-Light Deep Learning Architecture (UL-DLA) with GLCM and an SVM classifier, achieving 95.20% accuracy, benefiting from texture-based enhancement but limited by the shallow network depth. Ullah et al. (2022) explored Transfer Learning with pre-trained models, including InceptionResNetV2, ResNet, DenseNet, and MobileNetV2, achieving 94.65% accuracy. This result reflects improved feature transfer but shows slight inconsistencies due to varying layer complexities. More recently, Priya and Vasudevan (2024) introduced a Hybrid AlexNet-GRU model that obtained 96.00% accuracy, marking a notable advancement through the fusion of spatial and sequential feature learning. Likewise, Yildirim et al. (2023) proposed a Hybrid EfficientNetB0 + ShuffleNet + SVM approach that achieves 95.40% accuracy, emphasizing lightweight yet practical classification. The proposed TWFDM model achieved the highest accuracy of 96.25%, surpassing all existing methods. The performance gains of 0.25% over the Hybrid AlexNet-GRU and 0.6% over the CNN-Autoencoder model highlight the enhanced feature discrimination and robust learning capabilities of TWFDM. This consistent improvement demonstrates its statistical significance and validates that the proposed approach effectively captures multi-level tumor characteristics, resulting in more accurate and reliable brain tumor classification across all categories.

**Table 5:** Comparative analysis of TWFDM with the implementation of traditional methods.

Author & Year	Methodology	Accuracy (%)
Saeedi et al., 2023	2D CNN + CAE	95.65%
Rahman & Islam, 2023	PDCNN	94.25%
Qureshi et al., 2022	UL-DLA + GLCM + SVM	95.20%
Ullah et al., 2022	Transfer Learning (InceptionResNetV2, ResNet, DenseNet, MobileNetV2)	94.65%
Priya & Vasudevan, 2024	Hybrid AlexNet-GRU	96.00%
Yildirim et al., 2023	Hybrid EfficientNetB0 + ShuffleNet + SVM	95.40%
Proposed Method	TWFDM	1.1%

## 5 Conclusions and future scopes

This paper presents a brain tumor detection approach using a TWFDM that combines 2D-DCNN and 1D-DCNN. The TWFDM considers the original image as input to a 2D-CNN to capture spatial correlations among global-local attributes, and handcrafted features as input to a 1D-DCNN to characterize texture and shape attributes of brain MRI images. The proposed TWFDM provides the overall accuracy of 96.25%, recall of 96.34%, and precision of 96.31% and f1-score of 96.32% which has shown an imperative boost over the traditional techniques. In the future, the system’s efficiency can be improved by optimizing its hyperparameters to reduce the need for manual parameter tweaking. The effectiveness of the system can be validated for the real-time dataset to validate the generalization capability of the system.
